# Long intergenic non-coding RNA *GALMD3* in chicken Marek’s disease

**DOI:** 10.1038/s41598-017-10900-2

**Published:** 2017-08-31

**Authors:** Bo Han, Yanghua He, Li Zhang, Yi Ding, Ling Lian, Chunfang Zhao, Jiuzhou Song, Ning Yang

**Affiliations:** 1 Department of Animal Genetics and Breeding, National Engineering Laboratory for Animal Breeding, College of Animal Science and Technology, China Agricultural University, Beijing, 100193 China; 2 Department of Animal & Avian Sciences, University of Maryland, College Park, Maryland, 20742 United States; 3 Institute of Animal Science and Veterinary Medicine, Beijing Academy of Agriculture and Forestry Sciences, Beijing, 100097 China

## Abstract

Long intergenic non-coding RNAs (lincRNAs) are transcribed from non-coding DNA sequences. Studies have revealed that aberrant expressions of lincRNAs are associated with various types of cancers and neurological disorders. Marek’s disease (MD) is a highly contagious T-cell lymphoid neoplasia of chicken induced by Marek’s disease virus (MDV). In this study, we first identified and validated *linc-GALMD3* highly expressed in MDV-infected CD4+ T cells by RNA-Seq and qRT-PCR. By RNA-Seq analysis in MDCC-MSB1 cells after loss of function of *linc-GALMD3* by shRNA, we found that *linc-GALMD3* could positively *cis*-regulate its downstream gga-miR-223 gene expression. In contrast, it could *trans*-regulate the 748 differentially expressed genes (FDR < 0.01) that were mainly enriched into mitochondrial structure and cell cycle processes using GO analysis. Of these, the most significantly expressed gene *EPYC* might cause iris lesion in MD. The other eight genes, *NDUFA4*, *NDUFB6*, *NDUFV1*, *NDUFS8*, *SDHB*, *UQCRC1*, *UQCRC2*, and *COX7A2*, actively participated in oxidative phosphorylation in mitochondrial dysfunction and cell death. Most importantly, we found that the MDV replication was repressed when *linc-GALMD3* was knocked down in CEF cells. Our results suggested that *linc-GALMD3* might be a critical regulator in chicken MD and could be used as a candidate-promising mark for MD prevention, diagnosis, and treatment.

## Introduction

Although 93% human genome can be transcribed into RNAs, only 2% of these RNAs can be translated to proteins. The remaining 98% RNAs are basically non-coding RNAs (ncRNAs)^1, 2^. The ncRNAs are divided into small and long non-coding RNAs (lncRNAs) depending on the length of the RNAs^3^. Abundant lncRNAs were first found by the analysis of the mouse transcriptome in 2002^4^. LncRNAs are endogenous, longer than 200 nucleotides, and lack of open reading frame, which are ordinarily transcribed by RNA polymerase II^5, 6^. LncRNAs generally expressed low in organism, but have a tissue and cell-specific expression compared with the protein coding RNAs^7, 8^. Long intergenic non-coding RNAs (lincRNAs) are a kind of lncRNAs that transcribed from DNA sequences between coding genes^9^. So far, thousands of lincRNAs have been cataloged in human^10, 11^, mouse^11, 12^, zebrafish^13, 14^, and other species. More importantly, studies have revealed that aberrant expression of lincRNAs are associated with various types of cancers^15–24^ and neurological disorders^25–27^. However, the biological significance of lincRNAs in livestock animal diseases has not been extensively studied, especially in chickens^28^.

Marek’s disease (MD) was first described by Josef Marek^29^. MD is a highly contagious lymphomatosis induced by Marek’s disease virus (MDV), which is an alphaherpesvirus, gallid herpesvirus type 2 (GaHV-2)^30, 31^. MD is characterized by CD4+ T cell lymphoma formation, and MDV infection can be generally divided into four phases: early cytolysis (3–6 days post infection [dpi.]), latency (7–10 dpi.), late cytolysis (10–14 dpi.), and neoplastic lymphoma transformation (after 21 dpi.)^32^. In early cytolytic phase, the MDV meets its primary targets B cells, and later activated CD4+ T cells. The infected CD4+ T cells restrict the progeny virus produced by viral genome in latency, and serve as a target for transformation^33^. Syndromes are mainly occurred after infected with MDV, including acute MD causing death, classical MD having lymphomatous tumours in the skin, skeletal muscle, and visceral organs, neurolymphomatosis, ocular lymphomatosis, and immunosuppression^32, 34^. Thus, MD has leaded a huge economic loss to poultry industry^35^. At present, MD could be controlled by vaccine at one-day-old chicks. However, the MDVs are evolving and a new MDV strain with stronger virulence will emerge, further resulting in the loss of efficiency of the present vaccine. Previously, we have already identified more than 1,000 lincRNAs in chicken bursa, but the detailed study on lincRNA function is missing^36^.

In our previous RNA sequencing (RNA-Seq) work, *linc-GALMD3* was identified significantly highly expressed in MDV-infected chicken CD4+ T cells for two reciprocal cross chicken lines, which were line 6_3_ (MD resistant) × line 7_2_ (MD susceptible) and line 7_2_ × line 6_3_. In this study, the expression of *linc-GALMD3* was validated between MDV-infected and non-infected CD4+ T cells in these two reciprocal cross lines by qRT-PCR. In order to explore the biological function of *linc-GALMD3* in MD, we performed the RNA-Seq in MDCC-MSB1 cells after loss of function of *linc-GALMD3* by shRNAs, and the differentially expressed genes (DEGs) were analyzed after *linc-GALMD3* knockdown. In addition, the expressions of its up/downstream neighboring genes were examined by qRT-PCR in CD4+ T and MDCC-MSB1 cells. Furthermore, we used the GO enrichment and KEGG pathway analysis to explore the biological significance of *linc-GALMD3* in chicken MD.

## Results

### The identification of *linc-GALMD3* in chickens

**Figure 1:**
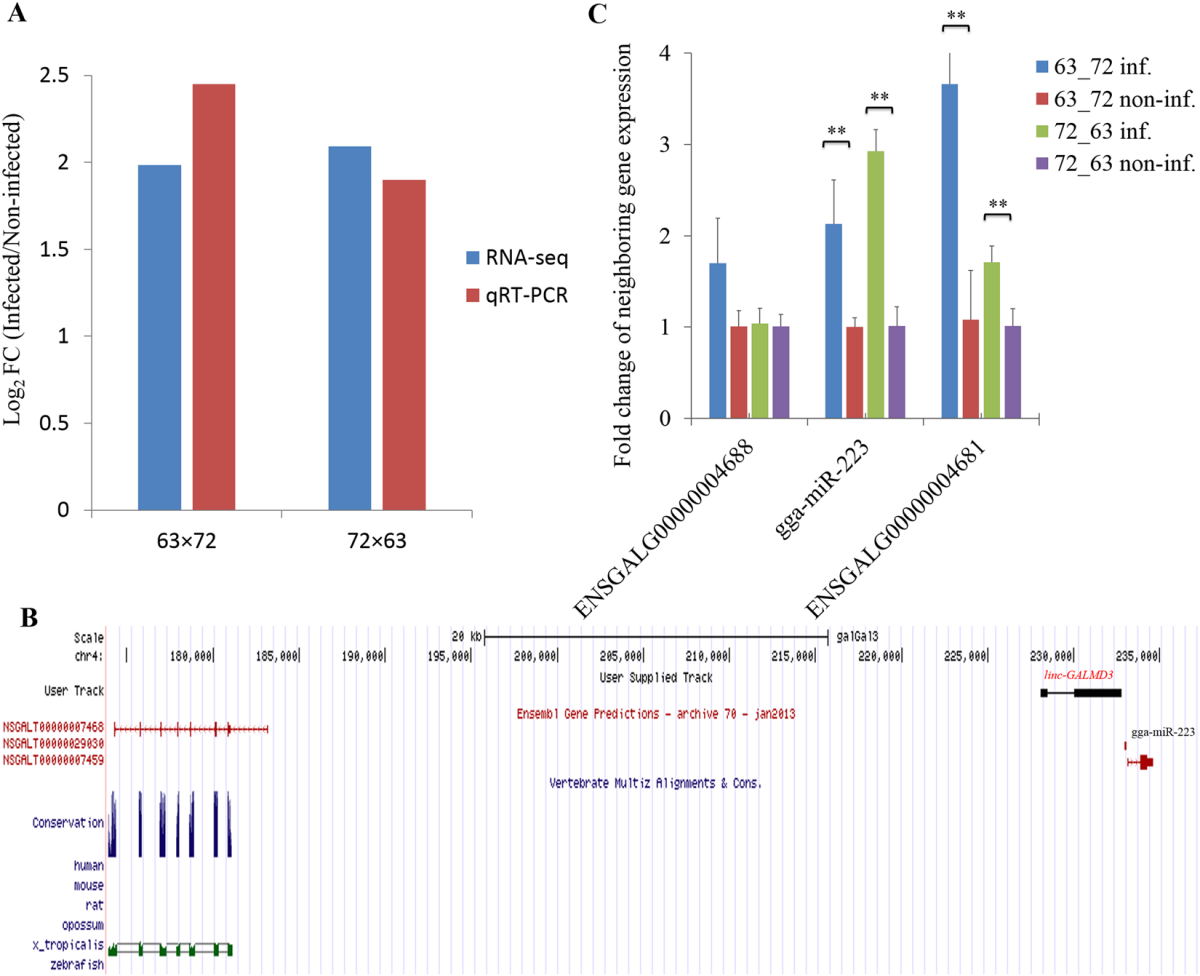
Identification and validation of *linc-GALMD3* and its neighboring genes. (**A**) Validation of *linc-GALMD3* expression. The mean value of log_2_ FC (Infected/Non-infected) was compared in the bar chart for CD4+ T cells from two reciprocal cross lines (6_3_ × 7_2_ and 7_2_ × 6_3_), respectively; FC means fold change; and the qRT-PCR data was normalized by *GAPDH* expression. Blue and red bars depict the RNA-Seq and qRT-PCR results, respectively. (**B**) Identification and conservation of *linc-GALMD3* among species. The black bar represented *linc-GALMD3*, NSGALT00000007468 and NSGALT00000007459 were the transcripts of upstream and downstream coding genes of *linc-GALMD3*, and NSGALT000000290030 was the transcript of the downstream gga-miR-223 gene. (**C**) Expression of *linc-GALMD3* neighboring genes in CD4+ T cells. Inf. means infected CD4+ T cells; non-inf. means non-infected CD4+ T cells; up/downstream gene expression were both normalized by *GAPDH* expression, ggg-miR-223 expression was normalized by chicken 5s rRNA expression, all the expression were normalized by the corresponding non-infection group; N = 3; and **p value < 0.01.

Previously, we identified that *linc-GALMD3* has the significantly higher expression in MDV-infected CD4+ T cells for two reciprocal cross chicken lines (6_3_ × 7_2_: fold change = 3.95, FDR = 0.0013; and 7_2_ × 6_3_: fold change = 4.26, FDR = 0.0014). In this study, we detected the expression of *linc-GALMD3* in CD4+ T cells in these two lines by qRT-PCR, and we found that the qRT-PCR result was consistent to that of RNA-Seq (Fig. 1A).

We also identified that *linc-GALMD3* has two transcripts (Fig. 1B and Supplementary Table S1). The *linc-GALMD3* was located between two protein-coding genes, ENSGALG00000004688 and ENSGALG00000004681, in chicken chromosome 4 (Galgal3), and ENSGALT00000029030 was a transcript of gga-mir-223 gene located at downstream of *linc-GALMD3*. The upstream neighboring gene was conserved between chicken and Xenopus tropicalis, however, no sequence conservation was found for *linc-GALMD3* and its downstream neighboring gene (ENSGALG00000004681) in human, mouse, rat, opossum, Xenopus tropicalis, and zebrafish (Fig. 1B), suggesting that *linc-GALMD3* was first discovered in chickens.

### *Linc-GALMD3* activates its downstream neighboring gene expression

The expression of up/downstream neighboring genes of *linc-GALMD3* were detected by qRT-PCR in MDV-infected and non-infected CD4+ T cells of the two reciprocal cross lines, and the expression of gga-miR-223 and ENSGALG00000004681 were significantly increased after MDV infection in both two lines (*p* value < 0.01), suggesting that the *linc-GALMD3* might activate the expression of its downstream neighboring genes through its *cis*-regulatory effects; however, the expression of the upstream gene, ENSGALG00000004688, has no obvious change (Fig. 1C; 6_3_ × 7_2_: *p*-value = 0.08; and 7_2_ × 6_3_: *p*-value = 0.82).

### The loss of function of *linc-GALMD3* in MDCC-MSB1 cells

**Figure 2:**
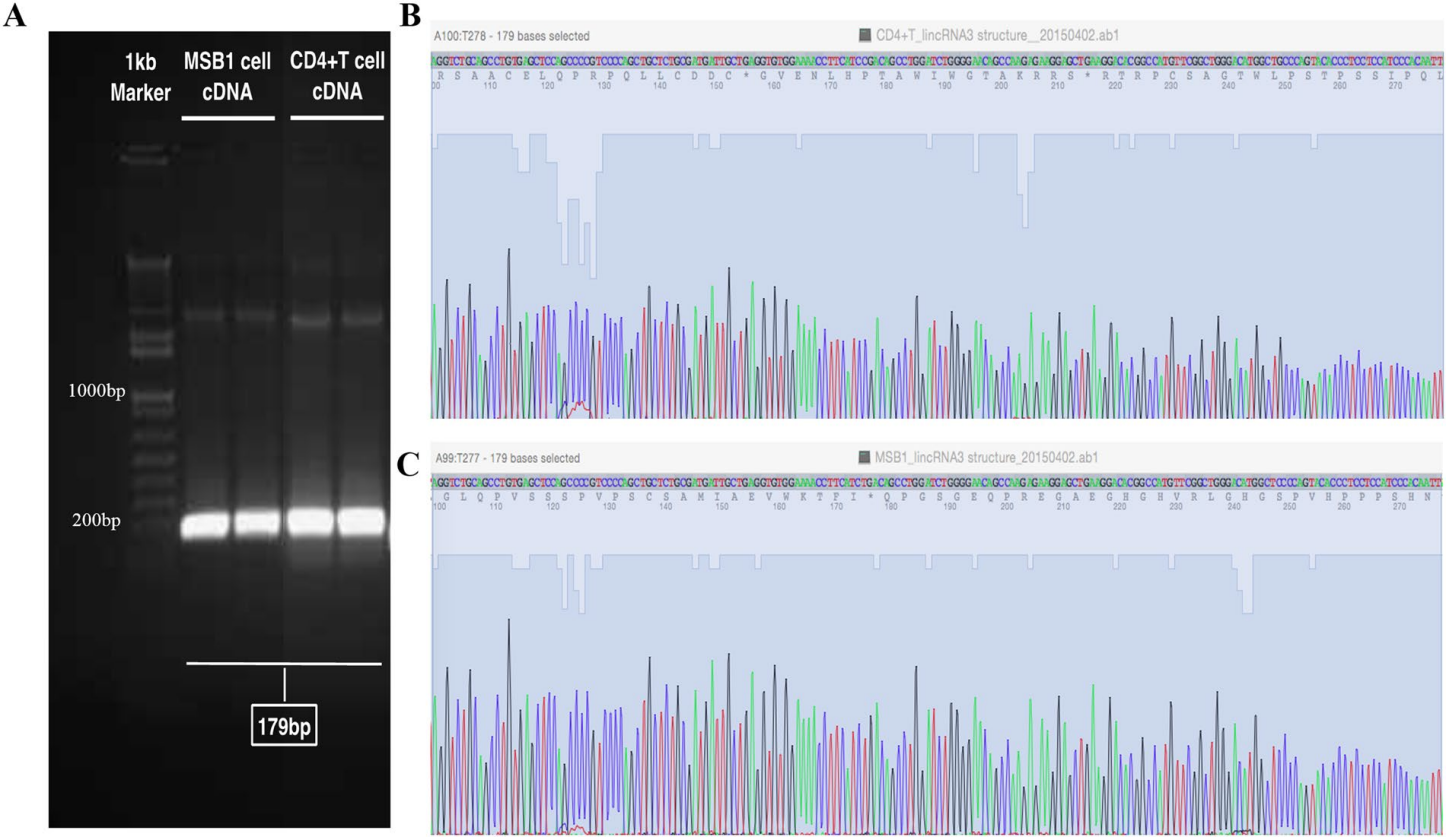
Confirmation of *linc-GALMD3* sequence in CD4+ T and MDCC-MSB1 cells by touchdown-PCR and Sanger Sequencing. (**A**) Product size confirmation using touchdown-PCR in MDCC-MSB1 and CD4+ T cells. Nucleotide sequences of *linc-GALMD3* in CD4^+^T (**B**) and MDCC-MSB1 (**C**) cells were respectively sequenced by Sanger Sequencing.

To confirm whether *linc-GALMD3* has the same transcript in chicken CD4+ T and MDCC-MSB1 cells, we designed the primer that spanned the introns to perform the touchdown-PCR and Sanger sequencing. We found the product lengths were totally the same in CD4+ T and MDCC-MSB1 cells (Fig. 2A). Additionally, Sanger sequencing results demonstrated that the size and nucleotide sequences of the touchdown-PCR product were exactly the same in CD4+ T and MDCC-MSB1 cells (Fig. 2B and C), which further confirmed that *linc-GALMD3* existed in CD4+ T and MDCC-MSB1 cells with the same transcripts.

**Figure 3:**
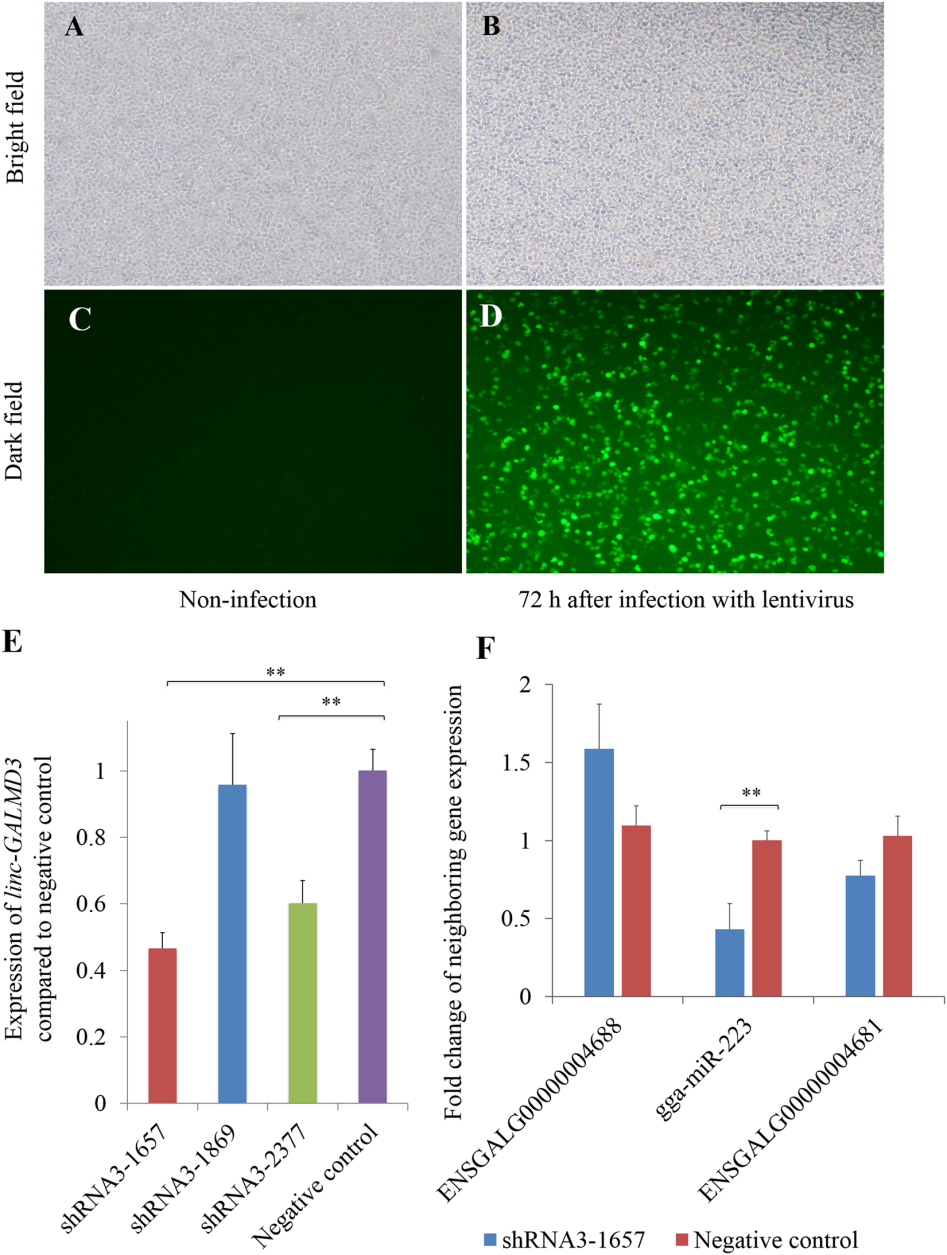
*Linc-GALMD3* knockdown by shRNAs in MDCC-MSB1 cells. The bright (**A**,**B**) and dark (**C**,**D**) field of non-infected (**A**,**C**) and MDC-infected (**B**,**D**) MDCC-MSB1 cells (×100) at 72 h after infection. (**E**) Expression of *linc-GALMD3* at 72 h after infected with shRNAs compared to the negative control group; N = 3; and **p value < 0.01. (**F**) Expression of up/downstream neighboring genes of *linc-GALMD3* after knockdown by shRNA3-1657; N = 3; and **p value < 0.01.

For *linc-GALMD3* knockdown, we designed three short hairpin RNAs (shRNAs) to interfere *linc-GALMD3* expression. The MDCC-MSB1 cells were infected by lentivirus that contains shRNA3-1657, shRNA3-1869, shRNA3-2377 or negative control (NC), and then the green fluorescence was observed at 72 h after infection. Results indicated that 70–80% of MDCC-MSB1 cells were infected by shRNAs or NC (Fig. 3D). As shown in Fig. 3E, the expressions of *linc-GALMD3* were decreased at 72 h when CEF cells were infected with three lentiviral shRNAs, respectively, compared with the negative control. Finally, shRNA3-1657 was chosen to do the further studies due to its highest interference efficiency.

Additionally, the expression of up/downstream neighboring genes of *linc-GALMD3* were measured after *linc-GALMD3* knockdown by shRNA3-1657, and the results showed that the gga-miR-223 expression was remarkably decreased after *linc-GALMD3* knockdown. The expression of downstream gene, ENSGALG00000004681, had an obvious reducing trend as well (Fig. 3F), indicating that the *linc-GALMD3* could positively regulate the expression of its two downstream neighboring genes, especially gga-miR-223 expression.

### RNA-Seq analysis after loss of function of *linc-GALMD3*

To investigate the global gene expression change after *linc-GALMD3* knockdown, we conducted RNA-Seq in MDCC-MSB1 cells with and without knockdown experiments. Total RNAs were extracted individually from MDCC-MSB1 cells after shRNA3-1657 or NC infection, and each group has three individuals for sequencing. After filtering out bad reads from raw data, each sample remained approximately 46 million high-quality clean reads (Q20 >= ~93%) with ~50% GC content, in which more than 74% of clean reads per sample were mapped to chicken genome (Galgal4). The details of data quality and mapping statistics were showed in Supplementary Table S2. Among all mapping reads, 32 million reads were aligned pairs with 69.57% concordant pair alignment rate. Results suggested that the RNA-Seq in this study has a high quality for further biological analysis.

**Figure 4:**
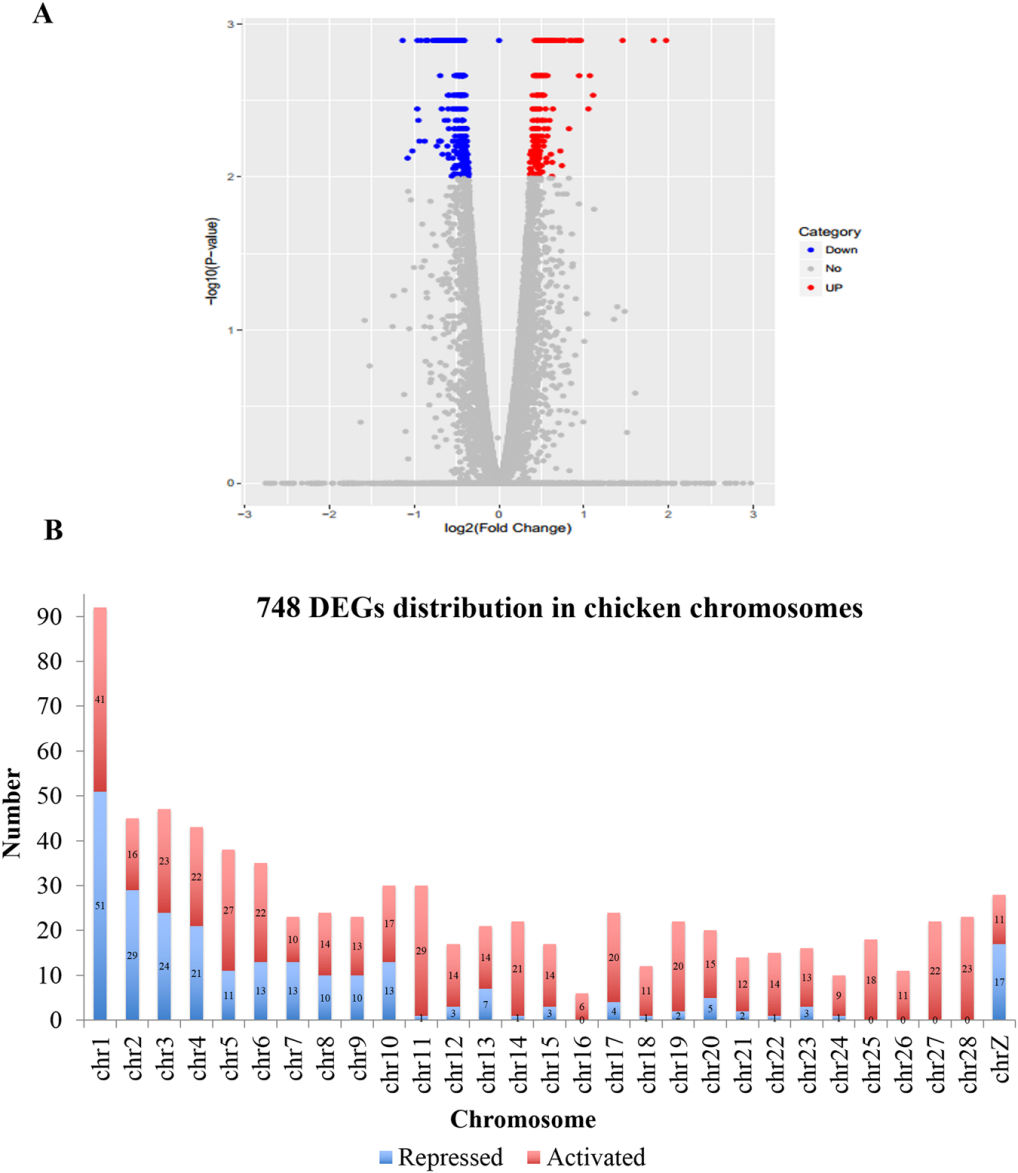
Profiles of differentially expressed genes (DEGs) after *linc-GALMD3* knockdown. (**A**) Volcano plot of 748 DEGs between *linc-GALMD3* knockdown and negative control groups. Red dots represent up-regulated DEGs and blue dots means down-regulated DEGs. (**B**) 748 DEGs distribution in chicken chromosomes. X-axis shows the chromosome number in chicken genome, and y-axis shows the number of DEGs in each chromosome.

In total, 748 genes were considered as differentially expressed genes (DEGs) after *linc-GALMD3* knockdown compared to the negative control, in which 246 were highly expressed when *linc-GALMD3* knockdown, and the other 502 genes were down-regulated (FDR < 0.01; Fig. 4A; Supplementary Table S3). Additionally, the 748 DEGs were equably distributed in every chicken chromosome, indicating that *linc-GALMD3* has driven its *trans*-regulation. Results also demonstrated that 67% DEGs could be activated by *linc-GALMD3*, and the remaining 33% DEGs might be repressed by *linc-GALMD3* (Fig. 4B). Among these, *EPYC* (Epiphycan, also known as PG-Lb; chr1: 43798642–43821219) was the most significant gene, which was highly conserved among human, rat, mouse, cow, frog, monkey, and zebrafish (see Supplementary Fig. S1).

### Validation of differentially expressed genes by qRT-PCR

**Figure 5:**
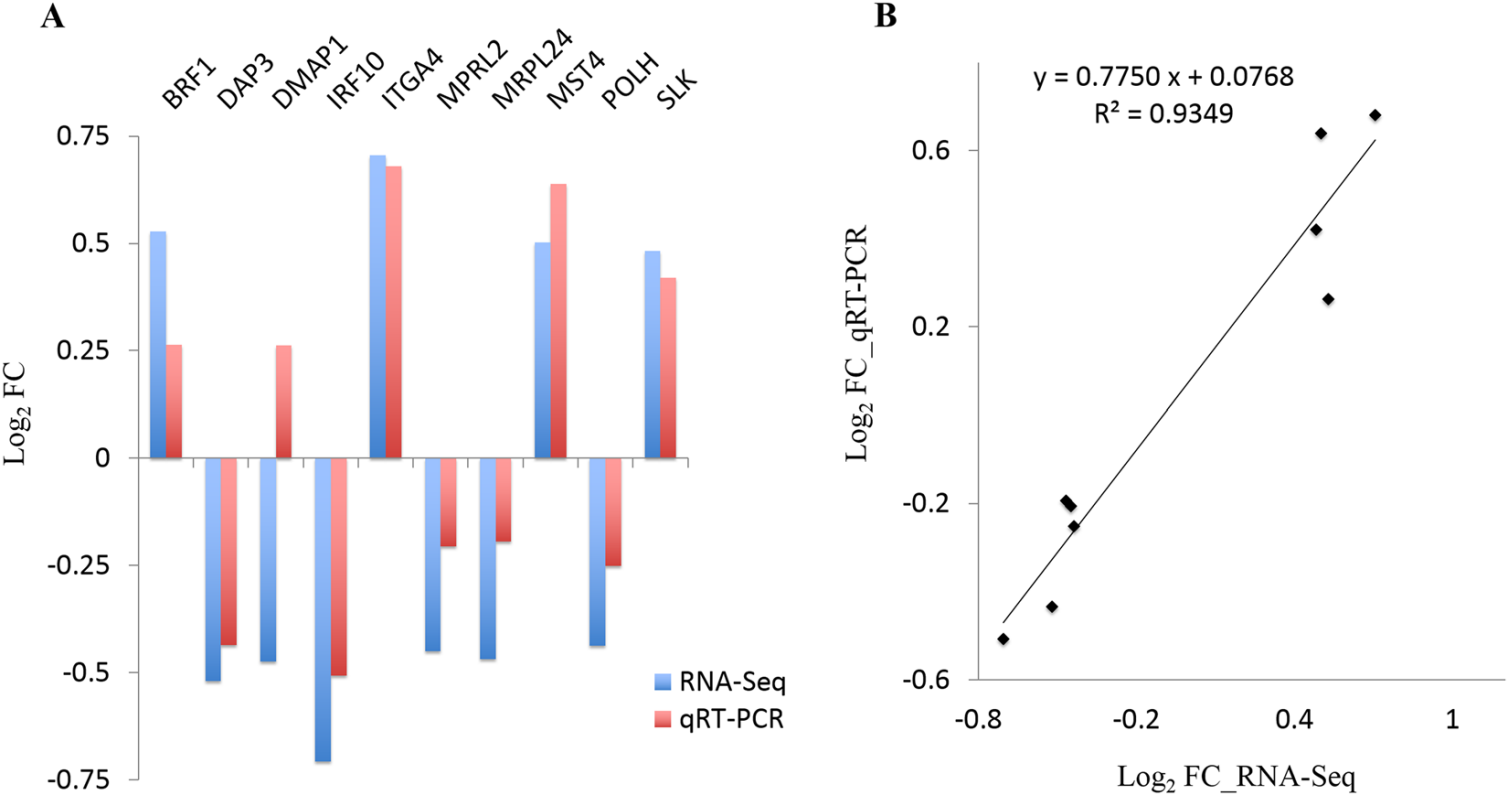
Validation of ten randomly selected differentially expressed genes (DEGs) by qRT-PCR. (**A**) X-axis represents 10 randomly selected DEGs for qRT-PCR, and y-axis represents the log_2_ (fold change = *linc-GALMD3* knockdown/negative control) derived from RNA-Seq and qRT-PCR, respectively. Blue and red bars depict the RNA-Seq and qRT-PCR results, respectively. (**B**) Regression analysis of the log_2_ (fold change = *linc-GALMD3* knockdown/negative control) values between RNA-Seq and qRT-PCR.

To confirm RNA-Seq results, ten DEGs were randomly selected to check their expression by qRT-PCR, and we found the results were consistent with RNA-Seq except *DMAP1* gene (Fig. 5). The expression level of four genes, including *BRF1*, *ITGA4*, *MST4*, and *SLK*, were notably higher after interfering *linc-GALMD3* expression; however, the expression of the other five genes were significantly down-regulated after *linc-GALMD3* knockdown (Fig. 5A). After removing *DMAP1* gene, the results of RNA-Seq and qRT-PCR showed a strong positive correlation with R^2^ = 0.9349 (Fig. 5B). Overall, the validations of these ten randomly selected genes by qRT-PCR confirmed the accuracy of the results of RNA-Seq.

### *Linc-GALMD3* drives its *cis* and *trans* regulation in chicken MD

A total of 751 target genes of gga-miR-223 were predicted by TargetScan 7.0 (see Supplementary Table S4), of these, 27 genes were also identified as DEGs after loss of function of *linc-GALMD3* (see Supplementary Table S5). Furthermore, 21 out of these 27 genes were up-regulated after loss function of *linc-GALMD3*, and previous results showed that the expression of gga-miR-223 was positively regulated by *linc-GALMD3* (Figs 1 C and 3F); therefore, these 21 DEGs might be released out when the expression of gga-miR-223 was decreased after loss of function of *linc-GALMD3*. The remaining six genes might be mainly modulated through *trans*-regulation of *linc-GALMD3*. These results indicated that *linc-GALMD3* could synchronously drive its *cis*- and *trans*-regulation in chicken MD.

### *Linc-GALMD3* activates the critical genes in mitochondrial dysfunction

**Figure 6:**
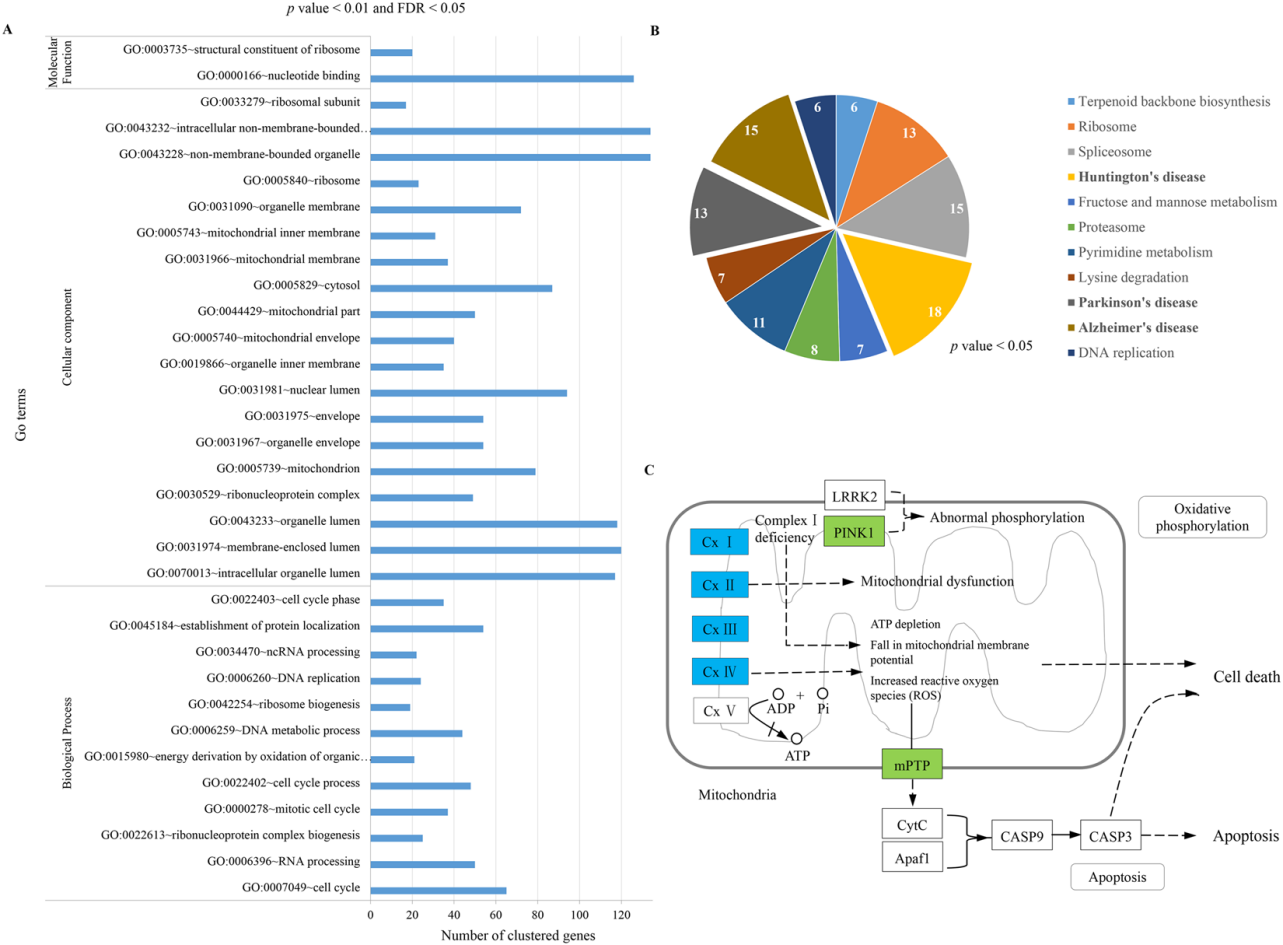
Biological function analysis for differentially expressed genes (DEGs) after loss function of *linc-GALMD3*. (**A**) Go Ontology (GO) analysis, *p* value < 0.01 and FDR < 0.05. (**B**) KEGG pathway analysis, *p* value < 0.05. (**C**) Mitochondrial dysfunction pathway. The genes in blue and green rectangles were significantly down-regulated after loss function of *linc-GALMD3*; Cx means protein complex.

DAVID Bioinformatics Resources 6.7 was used to explore the functional features of the 748 DEGs. Results showed that some DEGs were significantly enriched in biological processes, cellular components, and molecular functions (Fig. 6A; Supplementary Table S6; *p* value < 0.01, and FDR < 0.05). The most significant GO terms were: cell cycle in biological processes, intracellular organelle lumen in cellular components, and nucleotide binding in molecular functions. Moreover, one third of terms in biological process were enriched into cell cycle processes, and about one four terms in cellular component were clustered into the mitochondrial structures, suggesting that *linc-GALMD3* could mainly influence the cell cycle and mitochondrion structure related genes.

Additionally, the 748 DEGs were participated in 15 pathways (see Supplementary Table S6) and the most remarkably pathways were presented in Fig. 6B (p value < 0.05), including terpenoid backbone biosynthesis, ribosome, spliceosome, Huntington’s disease, fructose and mannose metabolism, proteasome, pyrimidine metabolism, lysine degradation, Parkinson’s disease, Alzheimer’s disease, and DNA replication. Of these, most of the DEGs were clustered into Huntington’s disease (18), Parkinson’s disease (13) and Alzheimer’s disease (15), which are all neurodegenerative diseases. Moreover, we found that eight genes were shared in these three pathways, including *NDUFA4* (NDUFA4, mitochondrial complex associated), *NDUFB6* (NADH: ubiquinone oxidoreductase subunit B6), *NDUFV1* (NADH: ubiquinone oxidoreductase core subunit V1), *NDUFS8* (NADH: ubiquinone oxidoreductase core subunit S8), *SDHB* (succinate dehydrogenase complex iron sulfur subunit B), *UQCRC1* (ubiquinol-cytochrome c reductase core protein I), *UQCRC2* (ubiquinol-cytochrome c reductase core protein II), and *COX7A2* (cytochrome c oxidase subunit 7A2) (see Supplementary Table S6). Otherwise, the eight genes were totally down-regulated after loss of function of *linc-GALMD3*, indicating that *linc-GALMD3* might sensitize them by *trans*-regulation in chickens (see Supplementary Table S3).

The eight genes encode the protein complexes in mitochondrial inner membrane, including complex I (*NADH: ubiquinone oxidoreductase*, contains *NDUFA4, NDUFB6, NDUFV1* and *NDUFS8*), complex II (*succinate dehydrogenase*, contains *SDHB*), complex III (*ubiquinol-cytochrome c reductase complex*, contains *UQCRC1 and UQCRC2*), and complex IV (*cytochrome c oxidase*, contains *COX7A2*), which are all participated in oxidative phosphorylation to cause mitochondrial dysfunction (Fig. 6C). The complex I deficiency and complex IV can lead to ATF depletion, loss of mitochondrial membrane potential, and the increasing of reactive oxygen species (ROS), thereby resulting in cell death. Moreover, a series of abnormal reactions above can also affect the expression of mPTP genes to cause cell death through apoptosis pathway, and the two mPTP genes, *VDAC1* (voltage dependent anion channel 1) and *VDAC2* (voltage dependent anion channel 2), were also found significantly decreased after *linc-GALMD3* knockdown (see Supplementary Table S3), indicating that *linc-GALMD3* could finally activate these two genes leading to apoptosis and cell death. Otherwise, the *PINK1* (PTEN induced putative kinase 1) gene was activated by *linc-GALMD3* (see Supplementary Table S3), and its protein can combine with LRRK2 to bring abnormal phosphorylation in mitochondria (Fig. 6C). These findings revealed that *linc-GALMD3* might activate the critical genes in mitochondrial dysfunction to cause apoptosis and cell death in MD.

### Suppression of MD viral replication after *linc-GALMD3* knockdown in CEF cells

**Figure 7:**
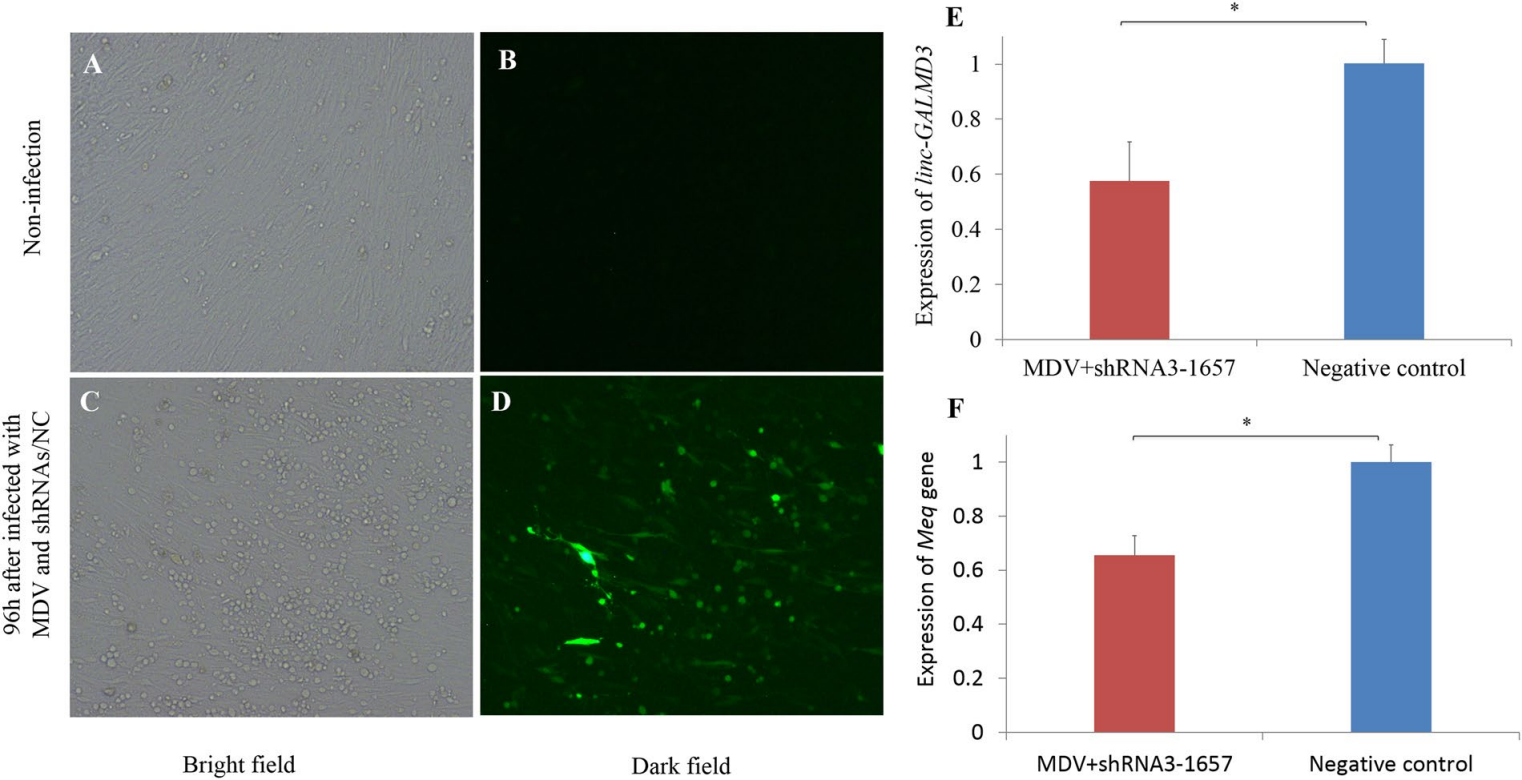
MDVs replication variation after loss of function of *linc-GALMD3* in CEF cells. The bright (**A**,**C**) and dark (**B**,**D**) field of non-infected (**A**,**B**) and infected (**C**,**D**) CEF cells (×100). The obvious cytopathic effect (**C**) and the green fluorescence (**D**) were observed in CEF cells at 96 h after infection with MDV and shRNAs/NC. (**E**) Expression of *linc-GALMD3* at 96 h after infected with MDV and shRNA3-1657 compared to the negative control group; N = 3; and *p value < 0.05. (**F**) Expression of *Meq* gene after *linc-GALMD3* knockdown by shRNA3-1657 in MDV-infected CEF cells; N = 3; and *p value < 0.05.

To investigate the biological function of *linc-GALMD3* in chicken, we infected the lentivirus containing shRNA3-1657 or NC into the MDV-induced CEF cells. In the bright field, the obvious cytopathic effect (CPE) showed up in the infected CEF cells (Fig. 7C), suggesting that the MDVs were in the reproduction processes. The green fluorescence was observed at 96 h after infection (Fig. 7D). The expression of *linc-GALMD3* was detected significantly down-regulated after infection with shRNA3-1657 compared to negative control (Fig. 7E; *p*-value = 0.01), and the Meq gene expression was significantly decreased after loss of function of *linc-GALMD3* (Fig. 7F; *p*-value = 0.04), showing that MD viral replication has been suppressed by decreasing the expression of *linc-GALMD3*.

## Discussion

### *Linc-GALMD3* first discovered and might stimulate chicken MD

To date, the domestic-animal lncRNA database (ALDB) has archived 8,923 chicken lincRNAs^37^. Muret *et al*. found 1,493 lincRNAs in chicken liver and/or adipose tissue, and considered that a novel lncRNA, *lnc_DHCR24*, was highly correlated with the *DHCR24* (24-dehydrocholesterol reductase) gene that encodes a key enzyme of cholesterol biosynthesis^38^. Previously, we identified more than 1,000 lincRNAs in chicken bursa, and considered that *linc-satb1* plays a critical role in MD immune response by regulating its nearby protein-coding gene *SATB1* ^36^. Here, we identified that *linc-GALMD3* was highly expressed in MDV-infected CD4+ T cells in chickens. Unlike protein-coding genes, the sequence conservation of lincRNAs was notably lower among species. Less than 6% zebrafish lincRNAs have the detectable sequence conservation with human or mouse lincRNAs^13^. The *linc-GALMD3* identified in this study showed no sequence conservation with other species, suggesting that *linc-GALMD3* was first discovered in chickens.

In mammals, lincRNAs are associated with various cancers, including prostate cancer^16^, breast cancer^18^, colorectal cancer^20, 21^, ovarian cancer^22^, and bladder cancer^24^, and also associated with disease progression, for example, the *HOTAIR* (HOX transcript antisense RNA) is highly expressed in the breast cancer and is a predictor for metastasis formation^39^, indicating that lincRNAs could be applied to the prediction and treatment of cancers. However, chicken lincRNAs were only reported to participate in MDV tumorigenesis^36^. The MD oncogene, *Meq*, was first identified in 1992, and played a critical role in MDV transformation and replication^40^. In this study, *linc-GALMD3* was identified highly expressed in MDV-infected chickens, showing that it might stimulate the chicken MD. Our validation assay showed that the MD viral replication was suppressed after loss of function of *linc-GALMD3*, indicating that the chicken MD might be alleviated by reducing the expression of *linc-GALMD3*. Certainly, whether *linc-GALMD3* can be used as a prevention and treatment tool for MD needs more validation and clinical trials.

### *Linc-GALMD3* activates gga-miR-223 expression

A rash of recent reports reveals that lncRNAs are powerful *cis*- and *tran*-regulators of gene activity^41, 42^. The most well-known nuclear lncRNA, *Xist*, has been reported to modulate gene expression in *cis* by acting as scaffolds for the recruitment of chromatin-modifying complexes and by altering the chromatin structure of target genes^43, 44^. The lncRNAs, *Air* and *Kcnq1ot1*, might recruit and silence specific *cis*-linked gene loci by interacting with chromatin and targeting repressive histone modifiers^45, 46^. Other *cis*-acting lncRNAs, such as enhancer RNAs, have shown to activate gene expression by locally regulating chromatin architecture^47^. Our study showed that the identified *linc-GALMD3* positively regulated the expression of its downstream gga-miR-223 gene through its *cis*-regulation.

MicroRNA-223 was reported to associate with cell growth and proliferation, many signal pathways, and various tumors^48–50^. It can regulate the expression of *IGF1R* in tumor tissue^51^, and modulate the cell proliferation by decreasing the *FOXO1* gene expression in human^52^. In chickens, gga-miR-223 have been shown to be involved in the immune organs development^53^, haemopoietic cell proliferation^54^, and macrophage differentiation^55^. Yao *et al*. investigated miRNA expression profiles using microarray, and found that several miRNAs, including gga-miR-223, were down-regulated in all MDV-transformed cell lines compared to normal splenocytes^56^. Otherwise, gga-miR-223 was found highly expressed in lungs of avian influenza virus infected broilers^57^. Moreover, Tian *et al*. utilized microarrays to screen miRNAs that were sensitive to MDV infection, and found that gga-miR-223 was significantly up-regulated in MDV-infected line 7_2_ compared with non-infected line 7_2_^58^, showing that gga-miR-223 was highly expressed in MDV-infected chickens, which was consistent with the high expression of gga-miR-223 in our MDV-infected chickens.

In addition, a report showed that the serum miR-223 might be a potential biomarker for Alzheimer’s disease evaluation^59^, and another study represented that miR-223/nuclear factor I-A axis could regulate glial precursor proliferation and tumorigenesis in the central nervous system^60^, indicating that miR-223 might play critical roles in neurological diseases. Therefore, we considered that gga-miR-223 with its target DEGs might be associated with neurologic lesions in MD.

### *Linc-GALMD3 trans*-regulates gene expression in chickens

In contrast, a growing number of lncRNAs, including lincRNA-*p21*, has been reported to regulate gene expression in *trans* by directing the chromatin localization of protein binding partners^61^ and by suppressing mRNA translation^62^. The lncRNA *HOTAIR* has shown to regulate expression of *HoxD* (homeobox D cluster) gene in *trans* by associating with chromatin modification complexes^10, 63, 64^. We used the RNA interference to silence the expression of *linc-GALMD3* in MDCC-MSB1 cells, which share properties of MD tumors and can be used as an excellent model for analyzing the molecular pathway and tumorigenesis of MD tumors^65^, and the subsequent RNA-Seq was used to uncover the changes of gene expression after loss of function of *linc-GALMD3*. Further, the qRT-PCR results for DEGs confirmed the reliability of the data analysis of RNA-Seq. Moreover, 748 DEGs were identified and equably distributed in chicken genome, which showed the *trans*-regulatory effects of *linc-GALMD3* in chicken MD.

### *EPYC* might lead to chicken iris lesion in MD

The most significant DEG, *EPYC* (*PG-Lb*), was originally isolated from chick embryo cartilage^66^. The protein encoded by *EPYC* is predominantly expressed in cartilage, and it regulates fibrillogenesis by interacting with collagen fibrils and other extracellular matrix proteins^67^. Haploinsufficiency of *EPYC* is associated with posterior amorphous corneal dystrophy, thereby resulting in the corneal opacification, thickness, and curvature, iris coloboma and atrophy, and iridocorneal adhesions^68^. Of note, MD can cause lymphocyte infiltration of the iris, pupils in unequal size, and blindness, and *EPYC* was currently identified down-regulated in MDV-chickens, so it was considered a candidate promising gene affecting iris lesion in MD chickens.

### *Linc-GALMD3* could affect mitochondrial dysfunction to cause chicken MD

As we know, MD is characterized by occurrence of T cell lymphoma and infiltration of organs and nerves by lymphocytes. The central nervous system is an important target organ for MDV, and the neurologic syndromes of chickens induced by MDV include classical transient paralysis (TP), acute TP, persistent neurologic disease and late paralysis^69^. The identified eight common genes encode the protein complexes in mitochondrial inner membrane and are participated in oxidative phosphorylation associated with mitochondrial dysfunction. The mitochondrial respiratory chain provides energy to cells by oxidative phosphorylation, and consists of four membrane-bound electron-transporting protein complexes (I-IV) and an ATP synthase (complex V). The *NDUFA4*, *NDUFB6*, *NDUFV1*, and *NDUFS8* are subunits of the multisubunit *NADH*. It was reported that the subunits of *NADH dehydrogenase* and *cytochrome c oxidase* (*COX*) were associated with chronic gastritis^70^. The *succinate dehydrogenase* subunits (*SDHB*, *SDHC* and *SDHD*) have been confirmed associated with pheochromocytomas and paragangliomas^71^. The *UQCRC1* was highly expressed in breast and ovarian tumors, and played a potential role in tumorigenesis^72^.

The relations between mitochondrial dysfunction and cancers were first investigated in 1930, when the researchers hypothesized that the increasing rates of aerobic glycolysis of various tumor cells might be due to the impaired respiratory capacity^73^. Since then, metabolic alterations associated with mitochondrial functions have been identified in multiply cancer cells^74, 75^. So far, the significance of ncRNAs in the field of mitochondrial epigenetics has rarely accomplished^76^. Mitochondrial diseases affect multiple organ system and show a tissue-specific manifestation^77, 78^. Researchers in the field of mitochondrial biology are increasingly discovering the regulatory interactions between mitochondrial function and ncRNAs biology^79^. LncRNAs have also been implicated in mitochondrial function, such as mitochondrial-associated *MDRL* ^80^, *CARL* ^81^, *TGFB2-OT1* ^24^, *UCA1* ^82^, and *RMRP* ^83^. Therefore, we considered that the *linc-GALMD3* might *trans*-regulate the expression of mitochondrial structure genes to affect chicken MD.

## Conclusion

In conclusion, we first discovered *linc-GALMD3* in chicken MD that could *cis*-regulate its downstream neighboring gga-miR-223 expression and *trans*-regulate gene expression on chicken genome, especially regulate the mitochondrial structure related genes. In addition, *EPYC* deficiency might cause iris lesion in MD. Meanwhile, we found that the repression of MDV replication was accompanied by decreasing *linc-GALMD3* expression, which implies that we may cure MD by blocking *linc-GALMD3* expression. Therefore, *linc-GALMD3* could be considered as a critical regulator in chicken MD and might be used as a candidate mark for MD prevention, diagnosis, and treatment.

## Materials and Methods

### Chicken lines and experimental design

Previously, the highly inbred Avian Disease and Oncology Laboratory (ADOL, East Lansing, Michigan, USA) line 6_3_ (MD resistant) and line 7_2_ (MD susceptible) experimental White Leghorn chickens were reciprocally crossed to produce 6_3_ × 7_2_ F1 population and 7_2_ × 6_3_ F1 population. For each F1 population, the chickens were divided into two groups with two individuals infected by MDV (vv+ strain, 648A passage 40) and two non-infected as controls. The MDV was injected intra-abdominally with 500 plaque-forming units (pfu) at the fifth day after hatching, and the peripheral blood was collected at 21 dpi. When blood was collected with anticoagulant (EDTA), CD4+ T cells was immediately isolated from peripheral blood mononuclear cells (PBMCs) by CD4+ T Cell Isolation Kit (MACS, Miltenyi Biotec, Bergisch-Gladbach, Germany). Briefly, PBMCs were incubated with Mouse Anti-Chicken CD4-PE antibody (Southern Biotech, AL, USA) and then incubated with Microbeads (Milteny Biotec) in a refrigerator for 15 min followed by washing and centrifugation steps. The supernatant was aspirated off completely. The cell pellet was re-suspended in MACS buffer, and then loaded into the column (Milteny Biotec) onto the magnet, and washed with MACS buffers. The columns were removed from the Separator and placed in clean tubes. MACS Buffer was added and cells were flushed out using a plunger. Finally, the CD4+ T cell pellet was obtained. In addition, the purity of the CD4+ T cells was determined by flow cytometry, and samples with more than 95% purity were used in the following studies. The animal challenge trials were followed according to guidelines established and approved by the USDA, ADOL Animal Care and Use Committee (ACUC) (April, 2005), and the Guide for the Care and Use of Laboratory Animals by Institute for Laboratory Animal Research (2011).

### RNA and DNA extraction

Total RNAs were isolated from cells using E.Z.N.A.^®^ Total RNA Kit II (Omega, GA, USA) as described by the manufacturer. Quality and quantity of the total RNAs were checked by 1% agarose gels, NanoPhotometer spectrophotometer (Implen, CA, USA) and Qubit 2.0 Flurometer (Life Technologies, CA, USA), and then they were reversely transcribed into cDNA by EasyScript First-Strand cDNA Synthesis SuperMix (TransGen Biotech, Beijing, China). DNA was extracted from CEF cells using TIANamp Genomic DNA Kit (TianGen Biotech, Beijing, China) as described by the manufacturer.

### Quantitative real-time PCR (qRT-PCR)

The qRT-PCR was performed by ABI 7500 system (Applied Biosystems, Foster City, CA, USA). Each reaction was in a final volume of 15 μL with 1 μL of cDNA/DNA, 0.2 μL of each primer (100 nM), and 1 × PCR Mix (Power SYBR Green PCR Master Mix, Applied Biosystems, Foster City, CA, USA). Primers for qRT-PCR were listed in Supplementary Table S7. The optimum thermal cycling parameters included 95 °C for 10 min, 40 cycles of 95 °C for 10 s, 60 °C for 1 min, 95 °C for 15 s, 60 °C for 30 s, and 95 °C for 15 s. All experiments were run in triplicate. The fold change of *linc-GALMD3* expression in qRT-PCR was calculated using the 2^−ΔCt^ method by comparing to *GAPDH* housekeeping gene between MDV-infected and non-infected CD4+ T cells. While, we used 2^−ΔΔCt^ method to measure the expression of *linc-GALMD3*, up/downstream neighboring genes, *Meq* gene, and *GAPDH* ^36^, chicken 5s rRNA^84, 85^, and *PCCA* (propionyl-CoA carboxylase alpha subunit)^86^ were the corresponding references. Data were expressed as the mean ± SD (standard deviation), and analyzed using a two-tailed Student’s *t*-test. The differences were considered to be statistically significant at *p* value < 0.05.

### Cell culture

MDCC-MSB1 provided by Dr. C. Itakura^87, 88^ is an MDV-transformed CD4+ T cell line. Cells were cultured in RPMI-1640 with 10% fetal bovine serum (FBS), under 5% CO_2_ at 37 °C. The primary chicken embryo fibroblast (CEF) cell culture was made from 10 day-old embryonated chicken eggs. The primary CEF cell monolayer was grown at 37 °C under 5% CO2 in 199 tissue culture medium supplemented with 10% FBS. All reagents for cell culture were purchased from Life Technologies (CA, USA).

### Touchdown-PCR

Touchdown-PCR was performed in a total volume of 10 μL, including 1 μL cDNA, 0.5 μL forward primer (10 μM), 0.5 μL reverse primer (10 μM), 5 μL Hot Start Mix (GoTaq^®^ Hot Start Green Master Mix, Promega, Madison, WI, USA), and 3 μL UltraPure^®^ Distilled Water (Invitrogen, Carlsbad, CA, USA). The primer sequences for confirming the structure of *linc-GALMD3* (*linc-GALMD3*_sequence) were listed in Supplementary Table S7. The optimal program was 5 min at 94 °C, 3 × (1 min at 94 °C, 1 min at 68 °C, and 2 min at 72 °C), 3 × (1 min at 94 °C, 1 min at (68-3i) °C [i = 1–5], and 2 min at 72 °C), 30 × (1 min at 94 °C, 1 min at 50 °C, and 2 min at 72 °C), with a final extension step of 10 min at 72 °C. PCR products were run on 1.5% agarose TAE (Tris-acetate-ethylenediamine tetraacetic acid) gels at 90 V for 1 h.

### Sanger sequencing

Target bands were cut from the gels through ultraviolet (UV). The DNA was extracted and purified using QIAquick Gel Extraction Kit (QIAGEN, Valencia, CA, USA). The purified DNA was ligated to T-Vector (pGEM^®^ T-Vector System Ι, Promega, Madison, WI, USA), and then transformed to DH5α competent cells (ZYMO Research, Irvine, CA, USA). The white colonies are positive and were screened after incubation at 37 °C overnight. Five white colonies were cultured in the shaker at 37 °C overnight. The plasmid DNA was isolated using Zyppy Plasmid Miniprep Kit (ZYMO Research, Irvine, CA, USA). The PUC/M13R primer (5′-CAGGAAACAGCTATGAC-3′) and BigDye Terminator v3.1 Cycle Sequencing Kit (Applied Biosystems, Foster City, CA, USA) were used for sequencing. Sanger sequencing was performed on ABI 3730 DNA Analyzer as described by the manufacturer.

### Short hairpin RNAs designed and knockdown efficiency detection

The shRNAs were designed and hypothesized by GenePharma Biotech (Shanghai, China). Three shRNAs for interfering *linc-GALMD3* and a negative control (NC) were respectively cloned to the shuttle vector (LV3-pGLV-h1-GFP-puro) labeled by GFP (green fluorescent protein), and then the vectors were packaged into lentivirus particles, respectively. The sequences of shRNAs were listed in Supplementary Table S8.

A total of 5 × 10^5^ MDCC-MSB1 cells per well were seeded into 24-well plates. Cells were infected with 40 μL lentivirus (2 × 10^8^ TU/mL) containing shRNAs or NC. The green fluorescence was observed through fluorescence microscope at 72 h after infection. The total RNA extraction and qRT-PCR were performed as described above.

CEF cells (4 × 10^5^ cells/well in 24-well plates) were co-infected with 2000 pfu MDV (CVI988, Beijing Lingyu Biological Technology Co., Ltd.) and 40 μL lentivirus (2 × 10^8^ TU/mL) containing shRNA3-1657 or NC. The cell lesion and green fluorescence were observed at 96h, subsequently the cells were harvested to isolate the total RNAs and DNA for qRT-PCR.

### RNA-Seq after *linc-GALMD3* knockdown

Total RNAs were extracted individually from MDCC-MSB1 cells after infected with shRNA3-1657 or NC. Each group has three individuals for the library construction. A total amount of 3 μg RNA per sample was used as input material for the RNA sample preparations. Sequencing libraries were generated using NEBNext® Ultra™ Directional RNA Library Prep Kit for Illumina® (NEB, USA) following manufacturer’s recommendations and index codes were added to attribute sequences to each sample. Briefly, mRNA was purified from total RNA using poly-T oligo-attached magnetic beads. Fragmentation was carried out using divalent cations under elevated temperature in NEBNext First Strand Synthesis Reaction Buffer (5X). First strand cDNA was synthesized using random hexamer primer and M-MuLV Reverse Transcriptase (RNaseH-). Second strand cDNA synthesis was subsequently performed using DNA Polymerase I and RNase H. In the reaction buffer, dNTPs with dTTP were replaced by dUTP. Remaining overhangs were converted into blunt ends via exonuclease/polymerase activities. After adenylation of 3′ ends of DNA fragments, NEBNext Adaptor with hairpin loop structure were ligated to prepare for hybridization. In order to select cDNA fragments of preferentially 150~200 bp in length, the library fragments were purified with AMPure XP system (Beckman Coulter, Beverly, USA). Then 3 μl USER Enzyme (NEB, USA) was used with size-selected, adaptor-ligated cDNA at 37 °C for 15 min followed by 5 min at 95 °C before PCR. Then PCR was performed with Phusion High-Fidelity DNA polymerase, Universal PCR primers and Index (X) Primer. At last, products were purified (AMPure XP system) and library quality was assessed on the Agilent Bioanalyzer 2100 system. The clustering of the index-coded samples was performed on a cBot Cluster Generation System using TruSeq PE Cluster Kit v3-cBot-HS (Illumia) according to the manufacturer’s instructions. After cluster generation, the library preparations were sequenced on an Illumina Hiseq 2500 platform and paired-end reads were generated.

### Data analysis and accuracy detection

Raw sequences were transformed into clean reads after certain steps of data-processing that includes removing the paired reads with adapters, removing the paired reads containing more than 10% of ‘N’ base in one single end and removing the paired reads containing more than 50% of low quality bases (sQ <=5) for each single read in one single end sequencing. All clean reads were aligned to the reference genome (Gallus_gallus-4.0/galGal4) by TopHat v2.0.9 and only concordant aligned pairs were considered. All aligned reads were assembled by cufflinks v2.2.1 and then merged into one GTF file by cuffmerge for differentially expressed analysis. Finally, the false discovery rate (FDR) of <=0.01 was applied as a threshold to call the genes with different expression levels by cuffdiff v2.2.1.

To validate the accuracy of sequencing and data analysis, we randomly selected ten DEGs for qRT-PCR, and the fold change of two groups was calculated after using 2^−ΔCt^ method to normalize to *GAPDH* housekeeping gene. The primers sequences were listed in Supplementary Table S7.

### Bioinformatics analysis

The target genes of gga-miR-223 was predicted using the online tools TargetScan 7.0 (http://www.targetscan.org). Gene Ontology (GO) and KEGG pathway analysis were performed after obtained the DEGs list through the online software DAVID Bioinformatics Resources 6.7 (https://david.ncifcrf.gov).

## Electronic supplementary material

Supplementary files

Supplementary Table S3 748 DEGs

Supplementary Table S4 Target genes of gga-miR-223

Supplementary Table S6 GO and KEGG pathway analysis
