# Supplementary material for: Long intergenic non-coding RNA GALMD3 in chicken Marek’s disease
Source: Sci Rep. 2017 Aug 31;7:10294. doi: 10.1038/s41598-017-10900-2 (PMC5579197; doi:10.1038/s41598-017-10900-2)
Supplement: Supplementary file 1 — Supplementary files [file 41598_2017_10900_MOESM1_ESM.pdf]

Supplementary Information

Long intergenic non-coding RNA *GALMD3* in chicken Marek's disease

Bo Han [1#], Yanghua He [2#], Li Zhang [3], Yi Ding [2], Ling Lian [1], Chunfang Zhao [1], Jiuzhou Song [2*], and Ning Yang [1*]

[1] Department of Animal Genetics and Breeding, National Engineering Laboratory for Animal Breeding, College of Animal Science and Technology, China Agricultural University, Beijing 100193, China.

[2] Department of Animal & Avian Sciences, University of Maryland, College Park, Maryland 20742, United States.

[3] Institute of Animal Science and Veterinary Medicine, Beijing Academy of Agriculture and Forestry Sciences, Beijing 100097, China.

[#] These authors contributed equally to this work.

[*] Corresponding Author:

Prof. Ning Yang (nyang@cau.edu.cn)

& Prof. Jiuzhou Song (songj88@umd.edu)

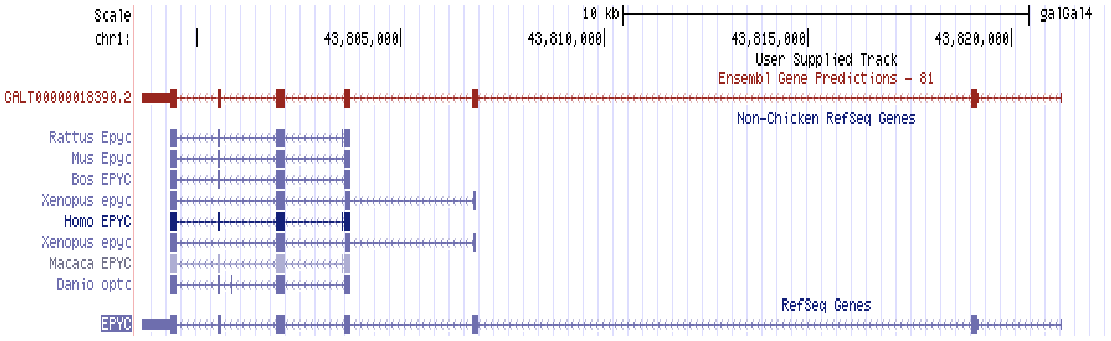

Supplementary Figure S1 Sequence conservation of *EPYC* in species. The GALT00000018390.2 was the transcript of *EPYC* gene in chickens. Rattus: rat; Mus: mouse; Bos: cow; Xenopus: frog; Homo: human; Macaca: monkey; and Danio: zebrafish.

Supplementary Table S1 Nucleotide sequence of *linc-GALMD3*.

gtgagtgcagagctggagggtgcagacacagcctgttctacctggagaacacagtcattgggaccgtgattcagtaatgaagatgatta
ggaattaatagtattaagtggtgtccataccttacttagtgctgggatgcaagtgtttcccctggctcctgcaggcacatgcaggcatgcta
cagatgggttactggctgtagcaagatcatgatctcaattagtcatagatgagacaccaatgaagagtgggggaaattgtgggatggag
gagggtgtactggggagccatgtcccagccgaacatggccgtgtccttcagctccttctcttggctgttccccagatccaggctgtcaga
tgaaggttttccacacctcagcaatcatcgcagagcagctggggacggggctggagctcacag

gctgcagacctgtctggtagccatgtaaaatcccccgagagatggtgagtgatttagggggggggaaaaccaaagctttcctaacttca
ataagggacttaattggagaatgcaggaagctctttctgtttcttccccagagtatgaagttgattgcttggtctgttttacagcgctaagctt
tcagttggaaatacccttctcagcttgaaaccagaagctctgacaatgacagatctattccgaggagataccatcgttacagggtggggt
agcaagctccattcccatctgctgatagaattatctccactgtgtgaccctcctcttatccgttgctgacagcaatgtttcttgtggctgttcc
ccccaccagctgcagtgagaggttattttcagctgccaggagcaatgctgtctctctgattttttttattttttttaatttttttactctcaaggcc
agatcttccagttagcaatcagcactgcaggtccgtggcagattcctacccagtctctgtacaacacctctgttgcatgttgatgattggag
tcaatgggggaagcttatttttttaccccgtgctctccacagtgtgcagtgttgaattcttacagaattacagaacattttcaggcagaggaa
tcccattctgcctttattcagactgacagcagctgtgtaaaccctctggggttcgttaatttttttttttttttttccatttcctgcagtacagcaga
gtgtgatttggaaggaagccatttggtccaccatgtacctgttttaattgcacttaaaccagtacatcttttgctagaatgaccttaaatcagc
ttattagcactgacacaagggttcttgaattatgcgcatttcatactttcacccattgcagcatactacttttatctgtggttgaaactcaccta
aattaggaggcagaacaaagagctgctcactatctacacctccctgaaagcctgatgcagagggcaggggaatgtaagacttgcaaa
gcctttgtagtaaccttgcctggcaattgattttatctttaaggctgtcgctgtttctttgctgtaaaatcctttttcactctactggttttggagct
catatgaaaagcacacctttggcacagatgtgccaccatcatttttaatttgtactttaatgtttgaaaaaacacaaagatgccctcttcagtt
tatttctgtgtgtaatgctgatggattcctgcaatcatccttgtaaaacagagtcctagagagatggaaaaggtttcatattgctcactgcag
aatgctgttaacacacctcagctaaattctgcctttgtgtctttaatggtcagccttccaaaatataattagaataaccacgtttggattttcatt
ttgataccaaaggtagctcagcttttctcagaaactgatgctgcaagtcatgtgaaatgaagatgaggggagcagccaggcagctaaaa
gcaccatgactcaaaccaggccatgcccagcatggcctgcagggctgtggtggacactggatctgaacgttatgaaagatgagtaca
gctcagtgcagctcaggaatcatgatgaatttctgagagagttgcacacttcagaattcaaagctgtttgaattctgaactgtcaacatccc
atctcagtaacagttggcaatatcatctcttgtgtccagtttggttaatgttctccagcggtttttttggaatttaattcccacttttgttaaaagga
acaggggagctggcagctggcacaaagctggcctttgtgcaaaggaataacgggagaagggggggtcatgtccatgcctaaaggaac
atggagaatgtccatgcaggcagcggagagctctgcttattgcaggtgagatttttttctgtgtgctggaaatggagcaggagggggggat
tcattcagcagcggaagagaaagtttcacttcattcccagagaagttccctgtttgagctgctctgcagagagtttttaacatcacagtcttt
ataatgagcagtgcagcttcctgctggtgggcagactctgggagccgagttgtgaggcagagttgtgcctttttccagataggcttgaag
ggttctcccttcaaatctttaggtttaggtaggaagatccactttaactattagtgtacaaaaagaggtaaaacacaaatcctgttcctgcca
ataggaactttggagtgcgtaaagccaggatttcaccccagcctgtgttccttgggatctgccagactggttacagcagaaatacatatct
agaactgggcactggaaagcaagtgaccacttgattgcaaaattaacagaggggcaaataaaagaaatgaagtccgagaagccccc
caaaactcgtttaagttcaagtggacagaaatgaatgtggtgagaagtctgctggaaaatgatgctcttatctctgccccgtgcagaattg
ttcccctgcagtatgttctctgggtctttgtctagacattgtgtgtctagagagatgtttgtcttcatgatgttgtacccaaatggcatgtctga
gtgctcagatggatgttttgtcaatatttcaagctaccaaagttcatctataacgaagggtgagtttagaactcagcattatttccagctccac
agagcttcagcatttcaacaaatagagcattttcctcagcagaaggcaacgtctgtc

Supplementary Table S2 Statistics for sequence quality and alignment information.

Sample_ID	Direction	Raw Reads	Clean reads	Clean Bases (G)	Error (%)	Q20 (%)	Q30 (%)	GC Content (%)	Mapped reads	Mapping rate (%)	Aligned pairs	Concordant pair alignment rate (%)
KD_1	Left	49053882	47307796	5.91	0.04	95.23	90.77	49.18	36060363	76.22	33644224	71.12
	Right	49053882	47307796	5.91	0.04	93.40	88.03	49.52	35587197	75.22		
KD_2	Left	49366405	47538317	5.94	0.04	95.33	90.91	49.95	35881837	75.48	33324664	70.10
	Right	49366405	47538317	5.94	0.04	92.98	87.33	50.30	35236220	74.12		
KD_3	Left	49170283	47332392	5.92	0.04	95.18	90.64	49.66	35848173	75.74	33383995	70.53
	Right	49170283	47332392	5.92	0.04	93.14	87.59	50.02	35303806	74.59		
NC_1	Left	47277132	45724877	5.72	0.04	95.14	90.55	49.89	34489406	75.43	32183836	70.39
	Right	47277132	45724877	5.72	0.04	93.16	87.48	50.26	33951982	74.25		
NC_2	Left	48147482	46551348	5.82	0.04	95.09	90.47	50.96	34149451	73.36	31830574	68.38
	Right	48147482	46551348	5.82	0.04	92.97	87.18	51.37	33557127	72.09		
NC_3	Left	47118918	45404446	5.68	0.04	95.06	90.38	51.55	32771154	72.18	30387451	66.93
	Right	47118918	45404446	5.68	0.04	92.55	86.49	51.92	32089361	70.67		
Average		48355684	46643196	6	0.04	94.10	88.99	50.38	34577173	74.11	32459124	69.57

KD: knockdown; NC: negative control; total clean reads number equals to sum of left and right-end reads; error rate: error rate of base; Q20: the percentage of bases with more than 20 Phred to the total of bases; Q30: the percentage of bases with more than 30 Phred to the total of bases; mapping rate (%) = (mapped reads/clean reads)*100; and concordant pair alignment rete (%) = (aligned pairs/clean reads)*100.

Supplementary Table S5 Differentially expressed target genes of gga-miR-223 after loss function of *linc-GALMD3*.

**Gene**	**locus**	**log_2(*linc-GALMD3* knockdown/NC)**	**p_value**	**q_value**
ABCF2	chr2:127172-139454	-0.446614	5.00E-05	0.00128047
ADNP	chr20:13752744-13772968	0.44269	0.00045	0.00627168
ARFIP1	chr4:33332207-33375924	0.420713	0.0004	0.00582238
ARID2	chr1:30694895-30801451	0.481215	5.00E-05	0.00128047
ARID4B	chr3:37438630-37524575	0.488664	5.00E-05	0.00128047
ERBB2IP	chrZ:20620963-20704566	0.536486	5.00E-05	0.00128047
FLAD1	chr25:1284085-1286863	-0.568204	5.00E-05	0.00128047
HIVEP2	chr3:52031851-52056523	0.587518	5.00E-05	0.00128047
HSP90B1	chr1:54869422-54879269	0.396712	0.00015	0.00291983
KIF16B	chr3:5413296-5548544	0.444856	0.0004	0.00582238
KMT2C	chr2:6528610-6717633	0.539796	5.00E-05	0.00128047
MBNL2	chr1:144576578-144648440	0.453142	5.00E-05	0.00128047
MICAL3	chr1:62010576-62110007	1.96944	5.00E-05	0.00128047
NUBP2	chr14:13202042-13206373	-0.43866	0.0001	0.00217374
PAOX	chr6:8918494-8927456	-0.456176	0.0001	0.00217374
PDS5B	chr1:173655058-173730298	0.442241	0.0007	0.00841129
PDZD8	chr6:28696247-28747446	0.486513	0.00025	0.00425936
PLEKHA1	chr6:30780611-30806669	0.419868	0.0002	0.00358643
RRP7A	chr1:49057125-49060762	-0.553294	5.00E-05	0.00128047
SLC25A6	chr4:16521843-16523820	-0.366419	0.00075	0.00879292
SP3	chr7:16580305-16610757	0.462307	5.00E-05	0.00128047
STAM	chr2:19520143-19541550	0.451644	0.0003	0.00483006
WWTR1	chr9:23264510-23304671	3.12886	5.00E-05	0.00128047
ZEB1	chr2:14306274-14411998	0.550936	5.00E-05	0.00128047
ZNF217	chr20:12871068-12883698	0.39107	0.0003	0.00483006
ZNF292	chr3:75929304-75982691	0.51515	0.00015	0.00291983
ZNF644	chr8:13554253-13606727	0.456592	0.0009	0.00986029

Supplementary Table S7 Primers used for qRT-PCR and touchdown-PCR.

Name	Direction	Sequence (5′-3′)
[a] *linc-GALMD3*_qRT-PCR	Forward	TCCCTGTTTGAGCTGCTCTG
	Reverse	AACTCTGCCTCACAACTCGG
[b] *linc-GALMD3*_sequence	Forward	AATTGTGGGATGGAGGAGGG
	Reverse	AGGTCTGCAGCCTGTGAG
[c] upstream neighboring gene	Forward	AGCAACCCAGGTTCCACTTT
	Reverse	AGGACTTGGACTTGGCGATG
[c] downstream neighboring gene	Forward	TACCAAAGTGGAGGGGGACA
	Reverse	GAAACCTCAGCTTCCGCTCT
[c] gga-miR-223	RT	GTCGTATCCAGTGCAGGGTCCGAGG TATTCGCACTGGATACGACGGGGTA TT
	Forward	GCGCTGTCAGTTTGTCA
	Reverse	GTGCAGGGTCCGAGGT
[#] Chicken 5s rRNA	RT	GTCGTATCCAGTGCAGGGTCCGAGG TATTCGCACTGGATACGACAAGCCT AC
	Forward	GCGCAATACCGGGTGCT
	Reverse	GTGCAGGGTCCGAGGT
[*] GAPDH	Forward	GAAGCTTACTGGAATGGCTTTCC
	Reverse	GGCAGGTCAGGTCAACAACAG
[d] MST4	Forward	TGATTTTGGAGTTGCTGGGC
	Reverse	CTCCCCTTTGGCTAGTTCGA
[d] IRF10	Forward	GAGCGGGAGAAGACCTACAA
	Reverse	TACTCCTCACCGAAGCACAG
[d] POLH	Forward	GCCCTCATCCAAAACTGCAA
	Reverse	TCGTGGAAGATGTTGCCTCT
[d] SLK	Forward	ACCTACGTTAGCACAGCCTT
	Reverse	TCTTCCGTGACTTCTGCCTT
[d] MRPL24	Forward	TGCTCAACCAGATCTCCCTG
	Reverse	GGCGTGTAGGTTTTGTCCAG
[d] MPRL2	Forward	CTGCCCTCCAAGAGACACAT
	Reverse	GGGGCAAGTTGACATAGCTC
[d] ITGA4	Forward	CCAGCACAGCAAGTCAGTAC
	Reverse	GATTCACACAGATGGCAGGC
[d] DMAP1	Forward	GCAGAGACAGACCACCTCTT
	Reverse	GGGATTTTCAGGTCAGTGCC
[d] DAP3	Forward	CCCCGTCGTCAGATATGTGA
	Reverse	CTGATCGAGCCTCTCCTTGT

[d] BRF1	Forward	TGCAGGCAAAACACCAACTT
	Reverse	AGGTAGAGACACGCAGCAAT
[e] Meq	Forward	GTCCCCCCTCGATCTTTCTC
	Reverse	CGTCTGCTTCCTGCGTCTTC
[&] PCCA	Forward	CAGACACACAGAGCCCATCTCT
	Reverse	TGGAGCAGTGGTGGCTGTT

[a] Primers for detecting the expression of *linc-GALMD3*;

[b] Primers for confirming the sequence of *linc-GALMD3* in CD4+ T and MDCC-MSB1 cells;

[c] Primers for up/downstream neighbouring genes of *linc-GALMD3* expression detection, RT means reverse transcription primer;

[d] Primers for detecting expressions of differentially expressed genes (DEGs) analyzed by RNA-seq after loss function of *linc-GALMD3*;

[e] Primer for detecting Meq gene in CEF cells after *linc-GALMD3* knockdown;

[*] Housekeeping gene for gene expression detection;

[#] Reference gene for miRNA expression detection;

[&] Reference gene for viral Meq gene expression detection.

Supplementary Table S8 Sequences of shRNAs for interfering *linc-GALMD3*.

shRNAs	Sequence
shRNA-3-1657	GCTGATGGATTCCTGCAATCA
shRNA-3-1869	GCTGCAAGTCATGTGAAATGA
shRNA-3-2377	GCAGCGGAAGAGAAAGTTTCA
Negative control (NC)	TTCTCCGAACGTGTCACGTTTC

Three shRNAs and NC were respectively cloned to the shuttle vector (LV3-pGLV-h1-GFP-puro) labeled by GFP (green fluorescent protein), and then the vectors were packaged into lentivirus particles (GenePharma Biotech, Shanghai, China), respectively.
